# Mechanisms underlying center of pressure displacements in obese subjects during quiet stance

**DOI:** 10.1186/1743-0003-8-20

**Published:** 2011-04-22

**Authors:** Francesco Menegoni, Elena Tacchini, Matteo Bigoni, Luca Vismara, Lorenzo Priano, Manuela Galli, Paolo Capodaglio

**Affiliations:** 1 Orthopaedic Rehabilitation Unit and Clinical Lab for Gait and Posture Analysis, Ospedale San Giuseppe, Istituto Auxologico Italiano, IRCCS, Piancavallo, Verbania (VB), Italy; 2 Neurology and Neurorehabilitation Unit, Ospedale San Giuseppe, Istituto Auxologico Italiano, IRCCS, Piancavallo, Verbania (VB), Italy; 3 Department of Neurosciences, Università di Torino, Torino (TO), Italy; 4 Dept. Bioengineering, Politecnico of Milan, Milan, Italy; 5 IRCCS "San Raffaele Pisana" Tosinvest Sanità, Roma, Italy

**Keywords:** balance, obesity, center of pressure

## Abstract

**Objective:**

the aim of this study was to assess whether reduced balance capacity in obese subjects is secondary to altered sensory information.

**Design:**

cross sectional study.

**Subjects:**

44 obese (BMI = 40.6 ± 4.6 kg/m^2 ^, age = 34.2 ± 10.8 years, body weight: 114,0 ± 16,0 Kg, body height 167,5 ± 9,8 cm) and 20 healthy controls (10 females, 10 males, BMI: 21.6 ± 2.2 kg/m^2^, age: 30.5 ± 5.5 years, body weight: 62,9 ± 9,3 Kg, body height 170,1 ± 5,8 cm) were enrolled.

**Measurements:**

center of pressure (CoP) displacements were evaluated during quiet stance on a force platform with eyes open (EO) and closed (EC). The Romberg quotient (EC/EO) was computed and compared between groups.

**Results:**

we found statistically significant differences between obese and controls in CoP displacements (p < 0.01) and no statistically significant differences in Romberg quotients (p > 0.08).

**Conclusion:**

the increased CoP displacements in obese subjects do not need an hypothesis about altered sensory information. The integration of different sensory inputs appears similar in controls and obese. In the latter, the increased mass, ankle torque and muscle activity may probably account for the higher CoP displacements.

## Introduction

In the last decade obesity has been recognized as a major world health problem characterized by an alarming growing rate and an important risk factor for various pathologies [1]. There is also epidemiological evidence that suggests that obesity increases the risk of falling [2] and complicates the treatment of the consequences [3-5]. Quite a number of studies have investigated the integrity of the postural control system in obese, specifically focusing on static posturography, by analyzing the centre of pressure (CoP) [6-12]. A general consensus emerges from the literature about an increase in CoP displacements in obese subjects. However, the physiological mechanisms underlying such generally observed behavior still need to be unveiled. In fact, the control of human stance depends on both the musculoskeletal and the nervous systems. The latter is strictly influenced by the integration of different sensory (i.e.: visual, vestibular and proprioceptive) inputs [13]. To our knowledge, this aspect (i.e.: the integration of different sensory inputs involved in the control of stance) has not been yet investigated in obese subjects. In this population, a condition that can lead to visual and vestibular alterations, known as "pseudotumor cerebri", has been reported [14]. An altered contribution of sensory endings and mechanoceptors has been recently proposed as a possible cause of the differences in CoP displacements between obese and healthy subjects [6]. Such hypothesis, however, has not been experimentally demonstrated. Rather, it has been formulated on the basis of previous findings about foot pressure distribution in obese individuals [10,15], and the role of mechanoceptors and cutaneous sensation in balance control [16,17].

Some neurological studies [18-21] have investigated the strategies of the central nervous system dealing with various sensory impairments. It appears that the system could adopt long-term plastic changes together with short-term gain modulations between the sensory modalities, depending on their availability and reliability. As a consequence, individuals with altered sensory inputs, and expectedly with greater CoP displacements, should place lower demand on the altered ("negative gain") and greater demand on the unaffected sensory inputs ("positive gain") to maintain postural stability. This mechanism has been previously defined as the "reweight of sensory inputs" [18].

Postural trials under eyes open (EO) and closed (EC) conditions and the so-called Romberg quotient (i.e.: EC/EO), extensively used in clinics, represent easy and non-invasive testing modalities to indirectly discriminate possible sensory impairments. In healthy subjects, the EO condition involves the integration of visual, vestibular and proprioceptive information, while under EC condition the subject relies on vestibular and proprioceptive inputs to maintain balance. Thus, the presence of altered sensory inputs yields different consequences on CoP displacements according to the EO or EC testing condition. For example, impaired vision may have two consequences: increased CoP displacements under EO (the system relies mainly on proprioceptive and vestibular information) and no changes under EC condition (the system relies again on proprioceptive and vestibular information). In such a case, the Romberg quotient would approximate 1 (i.e.: same performance in EC and EO), which is in line with the reports of two studies on individuals with vision loss [22,23]. As for impaired proprioception, two consequences are to be expected: possible increase of CoP displacements under EO (the system relies mainly on visual and vestibular information) and increased CoP displacements under EC condition (the system relies mainly on vestibular information). In this case, the Romberg quotient is expected to increase [24]. Similar consequences can be observed in individuals with impaired vestibular input, but they are not always detectable by the Romberg quotient [25,26].

Since sensory information is fundamental for balance control, we decided to focus our investigation on these aspects. Despite speculations had been made, to our knowledge, no studies have so far experimentally investigated the mechanisms underlying poor postural stability in obese subjects. Therefore the aim of this study was to assess whether the increased CoP displacements in obese subjects are secondary to altered sensory information.

## Materials and methods

### Subjects

Fourty-four obese subjects (Body Mass Index -BMI ≥ 30 kg/m2), 22 males and 22 females (BMI = 40.6 ± 4.6 kg/m^2 ^, age = 34.2 ± 10.8 years; body weight: 114,0 ± 16,0 Kg, body height 167,5 ± 9,8 cm), previously enrolled for another study [7], served as the obese group (O). All of them were free from conditions possibly associated to impaired balance: in particular, we decided to exclude subjects with vision loss/alteration, vestibular impairments, neuropathy, as detected by the clinical examination and those who reported symptoms related to intracranial hypertension [14]. Their lean counterpart consisted of 20 age-matched healthy subjects (H) recruited among the hospital staff (10 females, 10 males, BMI: 21.6 ± 2.2 kg/m^2^, age: 30.5 ± 5.5 years; body weight: 62,9 ± 9,3 Kg, body height 170,1 ± 5,8 cm). Subjects were naïve to the experimental protocol and procedures before the two proposed trials. All subjects included in the study had no evidences or known history of a gait, postural, or skeletal disorder and no history of falls. They were all sedentary subjects. The study was approved by the Ethic Committee of the Istituto Auxologico Italiano and an informed consent was obtained from each subject prior to participation.

### Experimental setup

Subjects were asked to look ahead with head straight, arms at the sides in a comfortable position and to stand barefoot on the force platform (Kistler, CH, sampling rate 100Hz), in a standard position with 30° feet abduction and heels at a distance of 8 cm. Two 60-second acquisitions were recorded: one under EO and another under EC condition [7].

No familiarization session before the trials was proposed to the subjects. A 2-minute interval time was provided between different trials. Three 60-second acquisitions under EO and 3 under EC condition were recorded. The mean value of the three trials under each conditions was calculated.

### Postural Parameters

Data from force platform were processed to obtain postural parameters about the CoP displacements. Specifically we computed following parameters in the antero-posterior (AP) and medio-lateral (ML) axes: Root Mean Square (RMS) of CoP positions (RMS_AP _and RMS_ML_), maximum excursion of CoP along the axes (RANGE_AP _and RANGE_ML_), and mean velocity of CoP displacements along the axes (MV_AP _and MV_ML_) [7].

For the planar movement of the CoP, we analyzed the RMS distance of the CoP series from the centre (RMS_CoP_), the area of the ellipse covering the 85.35% of CoP sway area (AREA_CoP_), as well as the mean CoP velocity (MV_CoP_) [7].

According to Rocchi et al. [27] and Chiari et al. [28], all parameters were normalized to the individual height in order to avoid the potential misinterpretation of data in between-groups comparisons. The Romberg index (EC score/EO score) was computed for all the parameters considered.

### Statistical analysis

Unless otherwise noted, all data are presented as mean ± one standard deviation, SD. Before using parametric statistical procedures, the assumption of normality was verified. Statistical analysis was performed using the Statistica software (StatSoft, U.S.). If the assumption of normality was verified, the parametric Student's t-test for independent groups was used to investigate differences between obese and lean subjects (p < 0.05), otherwise the non-parametric equivalent Mann-Whitney U-test was applied.

Comparisons of CoP parameters between healthy (H) and obese (O) groups under EO condition were performed in order to confirm the greater CoP displacements observed in obese individuals. Then we performed comparisons of Romberg quotients between O and H, and BMI-Romberg correlation by means of Pearson r coefficient, in order to assess the impairment of sensory inputs in obese subjects.

## Results

One male subject of the healthy group showed a Romberg value greater than 3.8 in 6 out of 9 parameters and was considered an outlier and eliminated from subsequent analysis.

In H, the EO parameters followed a normal distribution, while in O only MV_CoP _and RMS_CoP _did not violate the normality assumption. Results about differences between H and O in terms of EO parameters confirmed the grater displacements of CoP characterizing obese individuals (Table 1).

**Table 1:** Comparison of CoP parameters between healthy (H) and obese (O) groups.

*EO condition*	*H (n = 19)*	*O (n = 44)*	
RMS_AP _[mm]	3.0 ± 0.9	3.9 ± 1.0	§ p = 0.002
RANGE_AP _[mm]	16.7 ± 4.9	21.8 ± 6.0	§ p = 0.004
MV_AP _[mm/s]	6.5 ± 2.0	9.7 ± 1.5	§ p < 0.001
RMS_ML _[mm]	2.4 ± 0.6	3.1 ± 1.0	§ p = 0.008
RANGE_ML _[mm]	13.6 ± 3.9	17.8 ± 5.6	§ p = 0.005
MV_ML_[mm/s]	5.4 ± 1.6	6.9 ± 1.6	§ p = 0.001
RMS_CoP _[mm]	3.8 ± 1.1	5.0 ± 1.2	† p < 0.001
AREA_CoP _[mm^2^]	88.4 ± 42.5	143.7 ± 73.8	§ p = 0.005
MV_CoP _[mm/s]	9.4 ± 2.7	13.2 ± 2.1	† p < 0.001

§ Mann-Whitney U test; † Student's t-test. AP: anterior-posterior; ML: medio-lateral

In the H group, the Romberg quotient for weight and MV_AP _violated the normality assumption. In the O group, the Romberg quotient for RMS_CoP _, AREA_CoP_, RMS_AP_, and RMS_ML _did not violate the normality assumption.

We did not find any statistically significant difference between obese subjects and healthy controls, in terms of Romberg index of posture parameters (Table 2).

**Table 2:** Comparison of Romberg quotient of CoP parameters between healthy (H) and obese (O) groups.

*Romberg quotient*	*H (n = 19)*	*O (n = 44)*	
RMS_AP_	1.05 ± 0.23	1.18 ± 0.27	† p = 0.087
RANGE_AP_	1.07 ± 0.20	1.24 ± 0.37	§ p = 0.168
MV_AP_	1.17 ± 0.18	1.26 ± 0.22	§ p = 0.151
RMS_ML_	1.05 ± 0.20	1.06 ± 0.17	† p = 0.849
RANGE_ML_	1.14 ± 0.23	1.06 ± 0.26	§ p = 0.112
MV_ML_	1.13 ± 0.18	1.11 ± 0.18	§ p = 0.811
RMS_CoP_	1.05 ± 0.18	1.12 ± 0.20	† p = 0.141
AREA_CoP_	1.12 ± 0.38	1.27 ± 0.38	† p = 0.151
MV_CoP_	1.15 ± 0.17	1.20 ± 0.19	§ p = 0.323

§ Mann-Whitney U test; † Student's t-test

**Figure 1:**
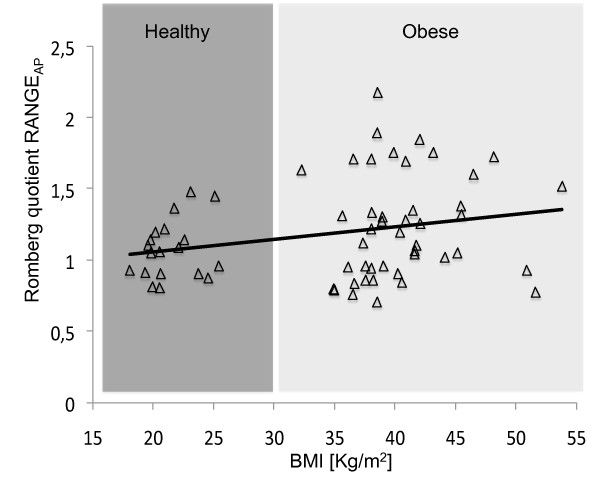
**Linear correlation between BMI and Romberg quotient about RANGE**_**AP**_**, with highlighted the classification between obese and healthy, and the regression line**.

As for the correlation between BMI and the computed Romberg quotients, despite statistical significance (p = 0.045), we found only a weak correlation (r = 0.253) between BMI and RANGE_AP _(Figure 1). All other parameters did not show significant correlation (r = 0.233 - 0.008, p = 0.066 - 0.953).

## Discussion

It is well known that the experimental conditions as well as a set of biomechanical factors have influence on stabilometric parameters: body height and weight, base of support area, maximum foot width, and feet opening angle [28,29]. Since the core of this study was the comparison between non-homogenous groups in terms of weight (H and O), we tried to minimize the influence of all factors but weight, by using the same experimental setup, experimental conditions, and by normalizing parameters to height. Our data show that under EO conditions obese individuals present higher CoP displacements during quiet stance than their lean counterparts.

CoP parameters can be classified as related to postural activity for maintaining stability (i.e.: velocity of CoP) (6) or related to effectiveness of the postural system (i.e.: magnitude of CoP displacements) [30]. In our study, obese individuals were characterized by an increased postural activity (Table 1, MV_AP_, MV_ML_, MV_CoP_). This did not lead to a reduction of CoP displacements, since the parameters related to the effectiveness of the postural system increased (Table 1, RMS, RANGE, AREA). Such findings are in line with previous studies [8,10-12], and supported by the correlation observed between body weight and CoP displacements during quiet stance [6,7,28].

Our main goal of was to investigate the mechanisms underlying reduced postural stability in obese subjects. In particular, whether the CoP strategy observed in obese patients could be due to altered sensory information. Since no statistically significant differences in Romberg quotient between O and H were found (Table 2), the integration of different sensory inputs appears to be similar in the two groups. Thus, our obese individuals report higher CoP displacements but do not seem to be characterized by sensory impairment. In fact, these results are not in accordance with those obtained in individuals with visual [22], proprioceptive [25,26], or vestibular impairments [30,31].

BMI could be considered an indirect measure of foot pressure [32]. If the hypothesis of an altered proprioception due to the increased foot pressure [6] was true, obese individuals should show greater balance impairment with an increased Romberg quotient as BMI increases. Our obese subjects appear to have grater sways, but only a weak correlation between BMI and the Romberg quotient of RANGE_AP _out of the nine parameters analyzed was found (Figure 1). This provides only limited evidence to speculate that foot pressure could thoroughly account for the differences in all the parameters analyzed between H and O groups.

Even if Romberg quotient could be not enough strength to explain effects of every single sensory input in balance control, our results do not support the hypothesis of the presence of altered proprioception in obese subjects and seem to back the findings of a neurophysiological study [33] in which non-diabetic obese people presented normal conduction velocity and latency but lower compound muscle action potential amplitude, probably related to the adipose layer. Moreover in the same study vibratory thresholds in obese subjects was not statistically different from non-obese controls, even if large standard deviation was found. Nevertheless, Romberg quotient has been used in several experimental settings and its quantification has been considered among parameters useful to detect alteration in postural stability.

Visual or vestibular impairments were excluded by the inclusion criteria and therefore CoP displacements in obese are likely to be not related to impaired sensory input.

It is known that balance depends on muscle activation and modulation and the correlation between CoP displacements and muscle activity has been shown [34,35]. Postural stability is optimal within a range of muscle activity: both very large and very small amounts of muscle activity lead to postural instability [36]. The increased body mass amplifies the ground reaction force (i.e.: mass times the acceleration of gravity), inducing higher torque at ankle level [37,38] and ultimately increasing muscle activity. Since muscle strength normalized per body mass is lower in obese than in their lean counterparts [39], greater amounts of muscle activity could be expected to preserve quiet standing, which may lead to a larger amount of stochastic activity and postural sway [35].

Such hypothesis is compatible with previous results [6,7] and with the results on the consequences of weight loss in obese subjects [8]. Even if the task chosen, quiet standing on two feet, may not have been challenging enough to elicit possible differences in the Romberg index between the groups, the proposed test was able to distinctly differentiate the two groups in terms of CoP displacements. Further complementary electromyographic recordings and foot pressure measurements are however needed to provide definitive evidence. Our patients did not show clinically detectable neuropathy, but future studies should include quantitative sensory testing to provide information about pre-clinical neuropathy, especially in obese subjects with altered quantitative insulin-sensitivity check index (QUICKI). We are aware that our study investigates a limited area of the physiological mechanisms involved in the control of human stance and the understanding of the whole dynamics related to balance control is still an open field of research and should take into account other factors than the ones presently considered.

However, we believe that such area plays a crucial role and our findings may generate potential rehabilitative spin-offs in the treatment of balance impairments and the prevention of falling.

## Competing interests

The authors declare that they have no competing interests.

## Authors' contributions

FM conceived the study and has made substantial contributions to its design, and interpretation of data and drafted the manuscript. ET has been involved in acquisition of data and analysis. MB has been involved in data and statistical analysis and revising the manuscript critically. LV has been involved in acquisition of data and analysis and statistical analysis. LP helped drafting the manuscript and revising it critically. MG has been involved in data and statistical analysis and revising the manuscript critically. PC conceived the study and drafted the manuscript revising it critically and has given final approval of the version to be published. All authors read and approved the final manuscript.

## Authors' information

**Paolo Capodaglio **received his M.D. degree from the University of Pavia, Italy, in 1988 and his specialization in Physical Medicine and Rehabilitation (PMR) from the same University in 1991. He was abroad for long and short visits (1992 University of Dusseldorf, 1995-1996 National Institute of Occupational Health, Copenhagen, Danemark) and thereafter developed collaborations with several foreign laboratories. At present, he is Head of the PMR Unit and the Laboratory for Research in Biomechanics and Rehabilitation at the Istituto Auxologico Italiano IRCCS in Verbania-Piancavallo, Italy and contract professor of PMR in the Medical School of the University of Brescia, Italy. He devoted most of his research to the functional evaluation in ageing and pathological conditions (spinal cord injuries, musculoskeletal disorders, obesity) and is reviewer for several indexed papers.
